# Using FIGO Nutrition Checklist counselling in pregnancy: A review to support healthcare professionals

**DOI:** 10.1002/ijgo.14539

**Published:** 2023-01-12

**Authors:** Sarah Louise Killeen, Niamh Donnellan, Sharleen L. O'Reilly, Mark A. Hanson, Mary L. Rosser, Virna P. Medina, Chandni Maria Jacob, Hema Divakar, Moshe Hod, Liona C. Poon, Lina Bergman, Patrick O'Brien, Anil Kapur, Bo Jacobsson, Cynthia V. Maxwell, Harold David McIntyre, Lesley Regan, Esraa Algurjia, Ronald C. Ma, Sumaiya Adam, Fionnuala M. McAuliffe, the FIGO Committee on Impact of Pregnancy on Long‐term Health and the FIGO Division of Maternal and Newborn Health

**Affiliations:** ^1^ UCD Perinatal Research Centre, School of Medicine University College Dublin, National Maternity Hospital Dublin Ireland; ^2^ School of Agriculture and Food Science University College Dublin Dublin Ireland; ^3^ Institute of Developmental Sciences University Hospital Southampton Southampton UK; ^4^ NIHR Southampton Biomedical Research Centre University of Southampton Southampton UK; ^5^ Department of Obstetrics and Gynecology Columbia University Irving Medical Center New York New York USA; ^6^ Department of Obstetrics and Gynecology, Faculty of Health Universidad del Valle, Clínica Imbanaco Quirón Salud, Universidad Libre Cali Colombia; ^7^ Divakar's Specialty Hospital Bengaluru India; ^8^ Helen Schneider Hospital for Women, Rabin Medical Center Petah Tikva Israel; ^9^ Sackler Faculty of Medicine Tel Aviv University Tel Aviv Israel; ^10^ Department of Obstetrics and Gynecology Prince of Wales Hospital, The Chinese University of Hong Kong Hong Kong Hong Kong SAR China; ^11^ Department of Obstetrics and Gynecology, Institute of Clinical Sciences Sahlgrenska Academy, University of Gothenburg Gothenburg Sweden; ^12^ Department of Obstetrics and Gynecology Stellenbosch University Cape Town South Africa; ^13^ Department of Women's and Children's Health Uppsala University Uppsala Sweden; ^14^ Institute for Women's Health University College London London UK; ^15^ World Diabetes Foundation Bagsvaerd Denmark; ^16^ Department of Obstetrics and Gynecology Sahlgrenska University Hospital Gothenburg Sweden; ^17^ Department of Genetics and Bioinformatics Domain of Health Data and Digitalization, Institute of Public Health Oslo Norway; ^18^ Maternal Fetal Medicine, Sinai Health and Women's College Hospital University of Toronto Toronto Canada; ^19^ Mater Health University of Queensland, Mater Health Campus South Brisbane Queensland Australia; ^20^ Imperial College London London UK; ^21^ The World Association of Trainees in Obstetrics & Gynecology Paris France; ^22^ Elwya Maternity Hospital Baghdad Iraq; ^23^ Department of Medicine and Therapeutics The Chinese University of Hong Kong Hong Kong Hong Kong SAR China; ^24^ Hong Kong Institute of Diabetes and Obesity The Chinese University of Hong Kong Hong Kong Hong Kong SAR China; ^25^ Department of Obstetrics and Gynecology, School of Medicine, Faculty of Health Sciences University of Pretoria Pretoria South Africa; ^26^ Diabetes Research Centre, Faculty of Health Sciences University of Pretoria Pretoria South Africa

**Keywords:** assessment, counselling, diet, FIGO Nutrition Checklist, nutrition, pregnancy

## Abstract

The period before and during pregnancy is increasingly recognized as an important stage for addressing malnutrition. This can help to reduce the risk of noncommunicable diseases in mothers and passage of risk to their infants. The FIGO Nutrition Checklist is a tool designed to address these issues. The checklist contains questions on specific dietary requirements, body mass index, diet quality, and micronutrients. Through answering these questions, awareness is generated, potential risks are identified, and information is collected that can inform health‐promoting conversations between women and their healthcare professionals. The tool can be used across a range of health settings, regions, and life stages. The aim of this review is to summarize nutritional recommendations related to the FIGO Nutrition Checklist to support healthcare providers using it in practice. Included is a selection of global dietary recommendations for each of the components of the checklist and practical insights from countries that have used it. Implementation of the FIGO Nutrition Checklist will help identify potential nutritional deficiencies in women so that they can be addressed by healthcare providers. This has potential longstanding benefits for mothers and their children, across generations.

## Synopsis

The FIGO Nutrition Checklist addresses key nutrition concerns for pregnancy. It can be used by healthcare professionals, or women themselves, to identify potential nutritional risks.

## 1 INTRODUCTION

Maternal nutrition is recognized as a high‐priority global health issue that requires urgent attention as it is integral to a variety of weight‐, nutrition‐, and health‐related Sustainable Development Goals (SDGs).^1^, ^2^, ^3^ Each year, weight‐related chronic diseases cause 4 million deaths. Globally, up to 800 million people are undernourished, and at least 1 billion people are deficient in micronutrients.^4^ Investment in maternal and child health, including nutrition, has long‐term benefits not only for population health, but also for the educational performance and economic productivity of the next generation.^5^

In many countries, there is a double or triple burden of malnutrition, characterized by concurrent high rates of overnutrition, undernutrition, and micronutrient deficiencies in the population, along with associated cardiometabolic or other health complications.^6^, ^7^ This burden is seen at an individual level where people with obesity or overweight may also have nutritional deficiencies such as iron and iodine.^8^ This challenge is also prevalent in maternal nutrition. Most women do not consume diets in line with the national dietary guidelines before or during pregnancy, and there are high rates of low or late nutritional supplement use.^9^, ^10^, ^11^ The COVID‐19 pandemic has further complicated this picture as it has been associated with reduced dietary diversity, increased calorie intake, and changes in food consumption.^12^, ^13^, ^14^, ^15^

Suboptimal diets and higher maternal weight during preconception and pregnancy can increase the risk of pregnancy complications and noncommunicable diseases in mothers and their children in the long term.^16^ Improving maternal nutrition is fundamental to improving child health outcomes, protecting women's health in the postpartum period, and potentially interrupting the intergenerational passage of poor health risk.^17^, ^18^ International organizations such as the World Health Organization (WHO) and FIGO recommend that all women receive nutrition and weight counselling during pregnancy.^19^ It is known that dietary counselling before and during pregnancy improves maternal nutrition knowledge, dietary intakes, and clinical outcomes like anemia, gestational weight gain, birth weight, with reduced risk of perinatal complications.^20^, ^21^, ^22^, ^23^, ^24^ Dietary counselling during lactation is also of value to support achievement of increased nutrient requirements during this time.^25^

A woman's engagement with health‐related material may differ depending on their pregnancy history or intention to conceive.^26^, ^27^, ^28^ Healthcare providers are encouraged to discuss pregnancy intention and provide nutrition information to women of childbearing age but engagement and practices around this vary.^29^, ^30^ Although preconception care provides an opportunity to address several risk factors before pregnancy, most women and couples may not access routine preconception care services.^31^, ^32^, ^33^ In addition, high rates of unplanned pregnancies seen globally act as a barrier to accessing this information.^34^ Studies have shown that women of childbearing age desire additional nutrition counselling, they consider nutrition during pregnancy important, and see clinicians as the most reliable supplier of this information.^35^, ^36^, ^37^, ^38^, ^39^, ^40^ Despite this, barriers exist such as a lack of nutrition training and supportive resources for healthcare providers.^41^, ^42^ Healthcare providers can use appropriate protocols and screening tools to identify nutritional risks and implement appropriate interventions.^43^, ^44^

**FIGURE 1:**
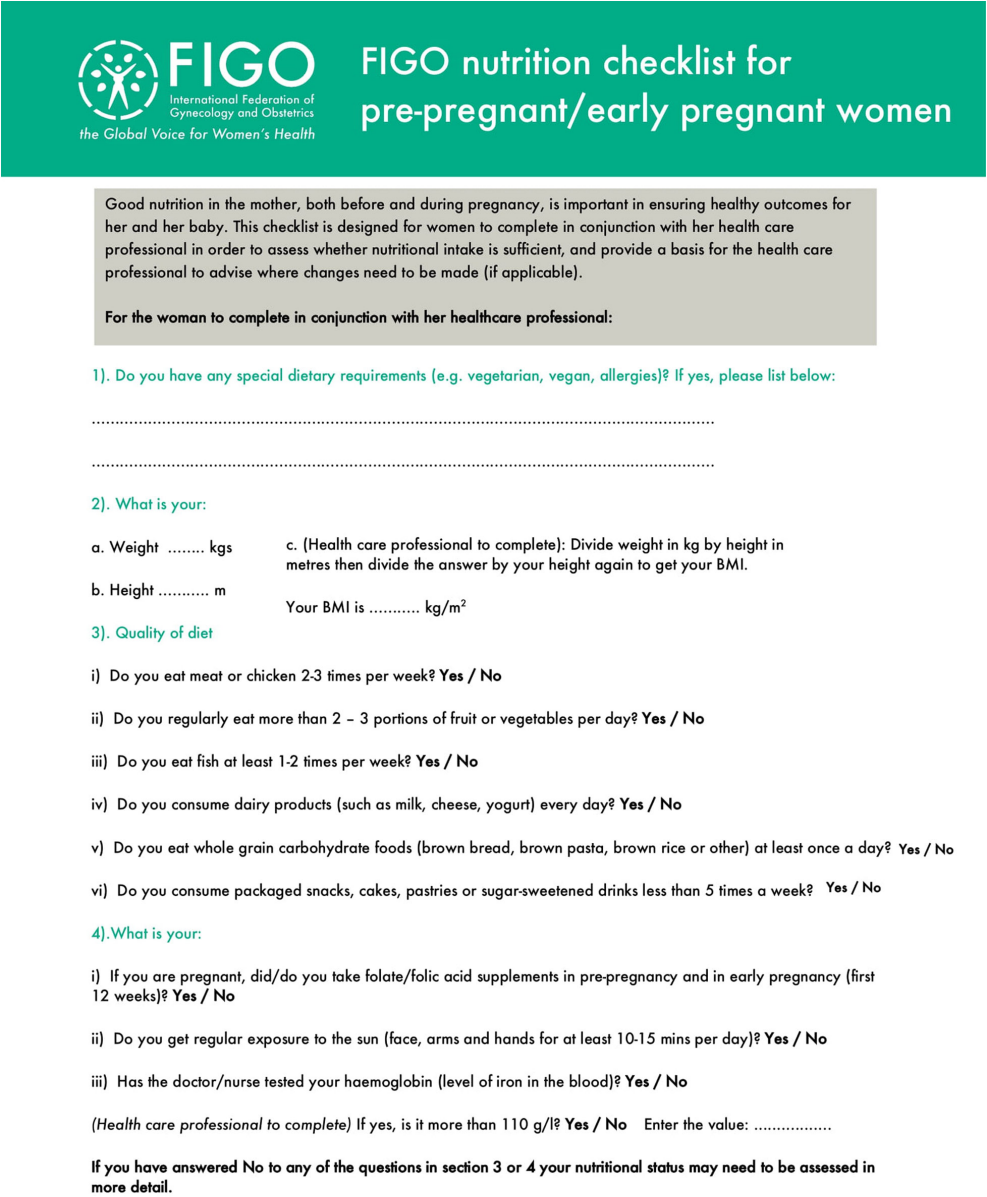
The FIGO Nutrition Checklist

The FIGO Nutrition Checklist is a validated tool that identifies unbalanced diets during the preconception, pregnancy, and postpartum periods (Figure 1). The checklist aims to facilitate conversations between healthcare providers and women on optimal dietary intakes.^45^ It can also be completed in advance of antenatal visits, thus saving time in clinical settings.^45^ Beyond this, the checklist can provide useful feedback to women on their dietary issues and fills a gap when nutrition counselling does not or cannot take place.^45^ The FIGO Nutrition Checklist collects information on dietary practices or ‘special diets’, body mass index, diet quality (number of servings or frequency of consumption of specific foods) and micronutrients (folic acid, vitamin D, and iron) (Figure 1). The back of the checklist (not shown in the figure, see Figure S1 includes evidence‐based information for healthcare providers based on FIGO's recommendations on adolescent, preconception, and maternal nutrition: ‘Think Nutrition First’^46^ and the US Institute of Medicine recommendations for gestational weight gain.^47^

Several studies supporting use of the FIGO Nutrition Checklist exist. In Ireland and Hong Kong, the FIGO Nutrition Checklist identified suboptimal diets in over 80% of women.^45^, ^48^ Tsoi et al.^48^ also found that the FIGO Nutrition Checklist was valid when compared with food frequency questionnaire data. Italian evidence suggests that the FIGO Nutrition Checklist was associated with pregnancy outcomes such as pregnancy‐associated plasma protein A and placental volume.^49^ Killeen et al.^45^ identified that most women found the checklist easy and quick to complete. Qualitative research on the FIGO Nutrition Checklist suggested a need for improved practices around nutrition counselling in antenatal care and a role for the checklist in meeting this need.^35^

The aim of this review is to provide an overview of international dietary recommendations for components of the FIGO Nutrition Checklist. A selection of freely available and published nutritional guidelines was reviewed for each component of the checklist.^50^, ^51^, ^52^, ^53^, ^54^ Countries were selected from Asia, Africa, Europe, Oceania, North America, and South America (Tables 1, 2). This review should be used as a guide only and local dietary and clinical guidelines should be followed where available. Beyond dietary guidelines, the review aims to provide practical recommendations and insights for implementation of the FIGO Nutrition Checklist. This review provides a practical tool for healthcare providers, public health specialists, and decision makers in antenatal care.

**TABLE 1:** Global recommendations for food‐based components of the FIGO Nutrition Checklist^a^

	Component of the FIGO Nutrition Checklist (daily consumption)			
	Fruit and vegetables	Dairy products	Wholegrains	Meat, poultry, or eggs
USA^50^	1.5–2 servings of fruit and 2.5–3.5 servings of vegetables per day	3 servings per day	3–5 servings per day	60–100 g per day (dependent on weight), 20%–25% of total calorie intake during pregnancy Consume with every main meal
India^51^	1–2 servings of fruit and 4–5 servings of vegetables per day	3–5 servings per day	50%–55% of total calorie intake 9 portions of 30 g per day	Variable, depending on dietary patterns
Ireland^52^	5 servings per day	3 servings per day	3–5 servings per day	2 servings per day in the first and second trimesters 3 servings per day in the third trimester
Canada^53^	Daily, consume a variety including dark green vegetables and orange vegetables	Daily. Drink fortified soy beverages if not drinking milk	Daily	Eat lean meats and alternatives daily
Australia^54^	At least 2 servings of fruit per day and at least 5 servings of vegetables per day	3.5 servings for those aged 18 years or under. 2.5 servings per day for those aged over 18 years	8 servings of carbohydrate foods per day (serving 30–40 g) for those aged 18 years or under, to 8.5 servings per day for those aged over 18 years. Choose mostly wholegrains	3.5 servings per day
Kenya^53^	5 servings per day	Everyday	With every meal	At least 2 servings per week. Eat red meat and liver when available
Colombia^53^	2–3.5 fruit servings 2–3 servings of vegetables	3–4 servings of dairy	3–3.5 servings of grains per day	2.5–3.5 servings of meat or poultry a day One egg per day

^a^ Information was gathered from various international sources as referenced. Where relevant, recommendations within guidelines were converted into grams per day to estimate servings.

**TABLE 2:** Recommendations for micronutrients addressed in the FIGO Nutrition Checklist^a^

	Folic acid	Vitamin D	Iron
USA^50^	400–800 μg at least 1 month before pregnancy and during the first 12 weeks	No specific recommendation	When recommended by a healthcare provider
India^51^	500 μg per day preconceptionally and throughout pregnancy	Only provide vitamin D supplementation if deficient	Supplementation of 100 mg elemental iron for 100 days during pregnancy from 16th week onward
Ireland^52^	400 μg per day throughout pregnancy, 5 mg for those at increased risk of neural tube defects	400 IU supplement per day plus dietary sources	Pregnancy 16–20 mg per day
Canada^53^	400 μg per day	600 IU per day	Potential benefits of 16–20 mg per day however no specific recommendation
Australia^54^	At least 400 μg per day	No specific recommendations	No specific recommendation
Kenya^53^	400 μg per day for 270 days during pregnancy	No specific recommendations	60 mg per day for 270 days during pregnancy
Colombia^53^	Use supplement (no dose/duration specification)	No specific recommendations	Use supplement (no dose/duration specification)

^a^ Information was gathered from various international sources as referenced. Where relevant, recommendations within guidelines were converted into grams per day to estimate servings.

## 2 SPECIAL DIETS DURING PREGNANCY AND LACTATION

A ‘special diet’ is a term used in the FIGO Nutrition Checklist to refer to a diet that varies from a traditional diet due to allergy, intolerance, or other medical needs, a religious or cultural diet, or a vegetarian or vegan diet. Women following special diets may be at risk of nutrient deficiencies such as protein and vitamin B12.^55^ Despite this, well‐planned special diets can be safe during pregnancy and lactation.^55^ Specific advice can be given to women who follow a special diet to address nutritional concerns highlighted by the FIGO Nutrition Checklist. Women following vegan diets may consume insufficient calcium and/or protein. Healthcare providers can screen for this, counsel on suitable alternative sources, and determine if supplementation is required.^56^ Some countries, such as India, have nutritional supplementation of key nutrients like iron and calcium as standard in antenatal care.^51^ In this instance, the FIGO Nutrition Checklist could be used to check compliance with supplementation guidelines.

## 3 FRUIT AND VEGETABLES

Fruit and vegetables are considered nutrient‐dense foods despite their low energy content.^57^ Polyphenols, oligosaccharides, and fiber found in fruit and vegetables are associated with a decreased risk of chronic diseases.^58^ Consuming fruit and vegetables in pregnancy is associated with a higher fiber diet that may help prevent glucose intolerance, pre‐eclampsia, and constipation.^59^, ^60^ Most countries recommend consuming at least five portions of fruit and vegetables per day (Table 1). Women with diabetes or altered glucose tolerance should be aware of high fructose consumption and its prenatal effects.^61^ Limited availability, cost of fruit and vegetables, poor tolerance due to nausea, reduced appetite, lack of social support, knowledge deficits, or cultural beliefs are barriers to reaching recommended fruit and vegetable intake.^62^, ^63^, ^64^

## 4 DAIRY

Dairy products are high in calcium, which provides numerous health benefits including reduced risk of type 2 diabetes and cardiovascular diseases.^65^ Globally, dairy products, cereals, vegetables, juices, and legumes are the main source of calcium.^66^ Other less bioavailable sources include fish bones, dried fruit, nuts, and seeds.^66^, ^67^ Low calcium intake during pregnancy increases the risk of pre‐eclampsia in calcium‐depleted women, stunted growth, and reduced peak bone density in teens of deficient mothers.^68^ Most countries recommend consuming 2–3 servings of dairy per day. Butter, ghee, and cream are dairy‐derived foods; however, they contain high amounts of saturated fat without calcium and are not recommended as part of this serving guide (Table 1). Pregnant women should avoid eating unpasteurized dairy products and soft cheeses.^69^ In cases where women do not consume dairy, fortified dairy alternatives such as soy, oat, or nut products, or plant foods such as dried fruit, nuts, and beans can be a source of key nutrients.^65^ There is also the option of supplementation for those at risk of calcium deficiency. In a multicenter trial of 500 mg calcium or placebo from before pregnancy to 20 weeks, women with over 80% adherence had significantly reduced pre‐eclampsia.^70^ In India for example, oral calcium supplementation is routinely advised to women during pregnancy.^51^

## 5 WHOLEGRAINS

Wholegrain consumption is associated with reduced risk of type 2 diabetes, cardiovascular disease, colorectal cancer, and obesity.^71^ The fiber content of wholegrains has the potential to assist in managing constipation, blood pressure, and blood glucose fluctuations.^72^ As illustrated in Table 1, the recommended amount of wholegrain consumption varies between countries. Some countries recommend having up to nine servings of grains, with half of these being wholegrains. These recommendations contrast with those from Ireland, for example, where it is recommended to consume 3–5 portions, depending on activity level (Table 1). The number of recommended servings varies, in part because the serving size is variable across different guidelines and reference documents (Table 1).

## 6 MEAT, POULTRY, AND EGGS

Adequate maternal protein intake promotes a healthy postnatal outcome and may influence childhood body composition.^73^ The proteins found in animal products are considered complete sources as they contain all the essential amino acids, whereas plant sources are incomplete but are considered complementary and complete when paired with another source.^74^ Plant proteins are becoming increasingly popular as potentially cost‐effective and sustainable protein options.^75^ Adequate protein intake during pregnancy is important as deficiency can cause complications such as miscarriage, fetal growth restriction, and reduced infant growth.^76^ Internationally, variations in guidelines are likely due to variability in serving sizes (Table 1). Protein requirements increase during pregnancy.^77^ In the USA, for example, the recommended daily intake of protein is 46 g per day (0.8 g/kg body weight/day) in the first trimester and 71 g per day (1.1 g/kg body weight/day) during the second and third trimesters.^78^ American guidelines for pregnancy recommend that women consume a variety of protein sources such as pulses, nuts, and fish.^50^ Recommendations in India state that the diet of pregnant women should contain an additional 0.5 g protein during the first trimester, 6.9 g during the second trimester, and 22.7 g during the third trimester of pregnancy.^51^

## 7 FISH

The nutritional health benefits of fish consumption primarily come from the long‐chain omega‐3 fatty acids eicosapentaenoic acid and docosahexaenoic acid. Fish also contain vitamins such as D and B2 (riboflavin), calcium, phosphorus, and minerals, such as iron, zinc, iodine, magnesium, and potassium.^79^ There is significant geographical variation in fish intake.^80^ The nutrients found in fish support the prevention of coronary heart disease, metabolic syndrome, and type 2 diabetes.^81^ They also provide neurocognitive benefits including IQ‐promoting benefits, communication, and other developmental outcomes.^82^ A diet containing adequate amounts of omega‐3 fatty acids is essential for fetal neurodevelopment and may protect against other adverse perinatal and longer‐term outcomes.^83^ Many countries recommend that pregnant women avoid consuming predatory fish such as shark and swordfish due to the risk of overexposure to mercury and other heavy metals.^84^ Given the benefits associated with fish consumption, moderate intake of fish, such as tuna, is considered safe when limited to no more than 1–2 times per week. The aim of this is to limit heavy metal exposure.^85^ Overall, most guidelines recommend consuming fish 1–2 times per week.^50^, ^51^, ^52^, ^53^, ^54^

## 8 PACKAGED SNACKS, CAKES, PASTRIES, OR SUGAR‐SWEETENED DRINKS

The guidelines for pregnancy and the general population are the same for processed, high sugar, high fat foods, which are that they are not recommended for consumption every day (Table 1). A Global Review of Food‐Based Dietary Guidelines found that most countries encourage people to limit salt; 89% to limit fat; and 84% to limit sugar; with 70% encouraging limiting all three.^86^ The aim of the checklist is to identify potential overconsumption of these foods and facilitate discussion around health‐promoting alternatives.

## 9 FOLIC ACID

Folic acid is the synthetic form of folate, a B vitamin naturally found in leafy green vegetables, citrus fruits, and liver.^87^ Deficiency increases the risk of neural tube defects in children.^88^ As a result, supplementation of 400 μg per day, paired with a healthy balanced diet, is recommended in many countries for women of childbearing age, regardless of their intention to conceive given the high rates of unplanned pregnancy worldwide.^31^, ^89^ The folic acid supplement can continue throughout pregnancy.^52^ Some women are at increased risk of neural tube defects, including those with obesity, and may require a higher dose (up to 5 mg/day) for at least the first 12 weeks of pregnancy^16^, ^52^ (Table 2). More than 40 countries have adopted mandatory folic acid fortification policies to prevent neural tube defects, and this may affect maternal levels.^90^ Folate and vitamin B12 deficiency can also cause anemia.^91^

## 10 VITAMIN D

Vitamin D is a fat‐soluble vitamin that plays an important role in calcium homeostasis and bone metabolism. Vitamin D can be obtained in the diet from a limited number of sources such as UV‐grown mushrooms, eggs, and fortified products.^92^ While it is produced by our skin, vitamin D deficiency is common.^93^, ^94^ Adult populations in Middle Eastern countries such as Iran and Syria have a very low average level of circulating vitamin D (14 ng/ml and 10 ng/ml, respectively),^94^ compared with adults in European countries like Denmark and France (26 ng/ml and 24 ng/ml, respectively).^95^ Several reviews have found a high prevalence of vitamin D deficiency even in countries with low latitude, where it was generally assumed that UVB radiation was adequate to prevent vitamin D deficiency, showing the potential benefits of supplementation and fortification.^96^ Foods fortified with vitamin D may contain approximately 100 IU per serving.^97^ Severe deficiency can lead to osteomalacia and rickets in both children and adults along with other adverse health outcomes.^98^ Saraf et al.^99^ found vitamin D deficiency, defined as 25‐hydroxyvitamin D (25[OH]D) levels below 50 nmol/L, in 54% of pregnant women globally. Although poorly defined in many regions, the breakdown by WHO region varied from 87% in Southeast Asia, 83% in the Western Pacific, 64% in the Americas, 57% in Europe, and 46% in the Eastern Mediterranean.^99^ The high prevalence of maternal vitamin D deficiency may be related to differences in ethnicities and/or lifestyles (sun exposure, dietary intake, skin melanin, wearing veiled or covered clothes) rather than increased physiological requirements.

## 11 IRON

Iron deficiency anemia is one of the most common health problems in women of reproductive age, affecting over one‐third of pregnant women globally.^100^ This anemia results in adverse outcomes such as increased maternal and infant mortality, mental and physical development issues, and impaired cognitive function in newborn babies.^101^ Dietary interventions are more effective in the long term for prevention of iron deficiency anemia than supplementation and have advantages in relation to compliance, long‐term acceptability, and cost‐effectiveness.^102^ In high‐risk populations however, supplementation may be more effective at reaching guideline‐specified optimal levels of iron in the diet.^103^ The FIGO Nutrition Checklist addresses iron intake and anemia screening. In relation to diet, it asks about the intake of meat, poultry, fish, vegetables, and fruit. Of these, red meat and other meat or fish are sources of the highly bioavailable heme iron. Other sources of iron include egg yolks, dark green leafy vegetables, beans, peas, dried fruit, and fortified cereals, although these are predominantly non‐heme iron, which is a less bioavailable form.^104^ Consuming iron‐containing foods alongside those with vitamin C, such as citrus juice or fruit or vegetables, may enhance iron absorption.^103^ Similarly, those at risk of deficiency should avoid eating foods that will inhibit iron absorption with the iron‐containing food. These include foods that are high in calcium, tannins, or phytates.^103^ The recommendations for iron vary between countries, with some including supplementation (Table 2). A morning dose of supplement may promote optimal response and alternate days could be considered to reduce gastrointestinal symptoms.^105^

## 12 FUTURE DIRECTIONS FOR THE FIGO NUTRITION CHECKLIST

**FIGURE 2:**
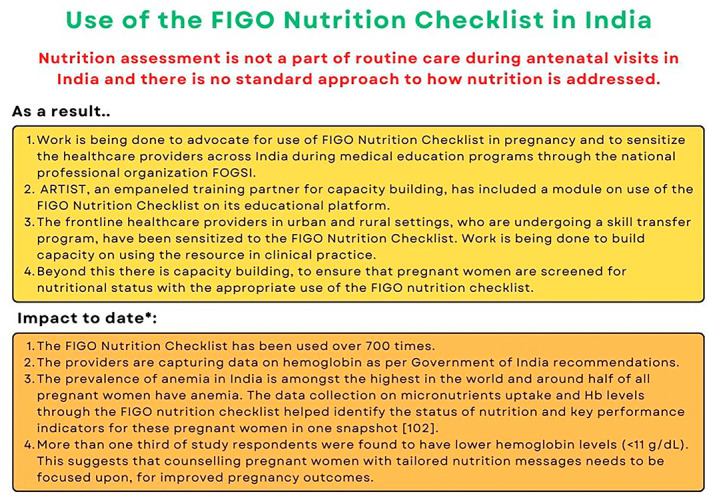
Use of the FIGO Nutrition Checklist in India. Abbreviations: FOGSI, Federation of Obstetric and Gynaecological Societies of India; ARTIST, Asian Research and Training Institute for Skill Transfer. *Data unpublished

**FIGURE 3:**
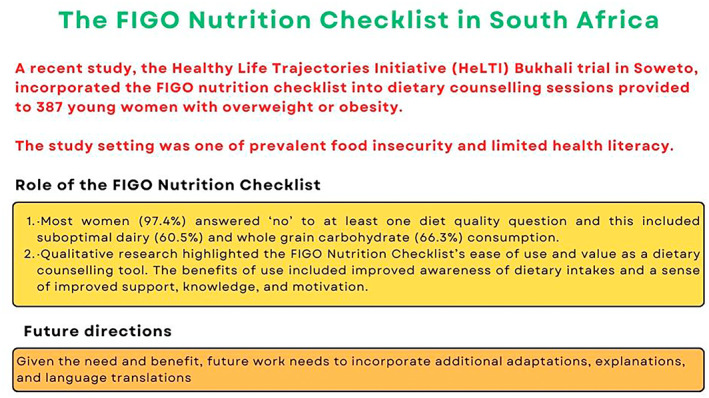
The FIGO Nutrition Checklist in South Africa. Full data available from Soepnel et al.^115^

**FIGURE 4:**
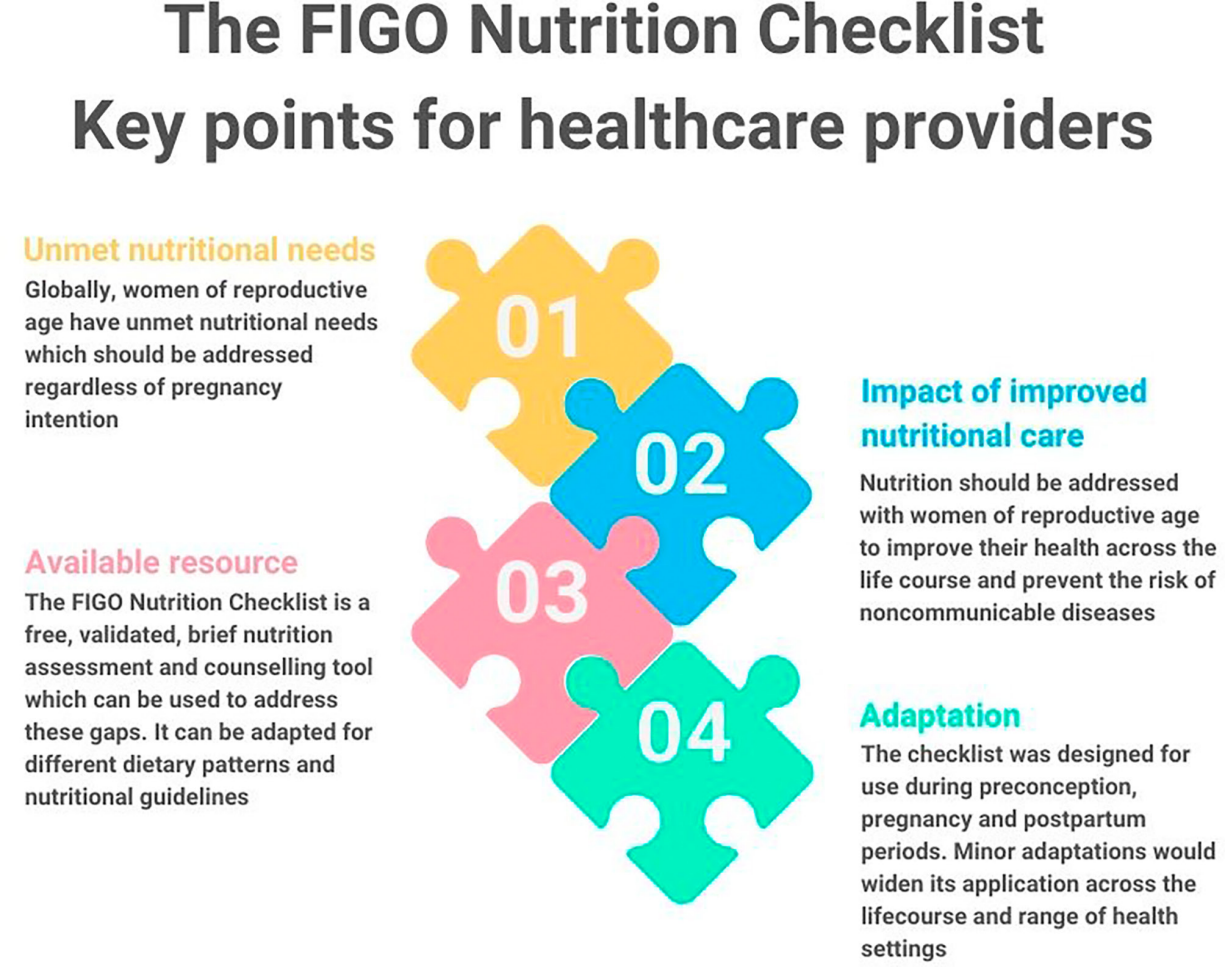
Key messages for healthcare providers

Disseminating health messages can be an effective way of educating people.^106^ Barriers that prevent women from improving their diet include differing priorities, income, and cultural norms.^107^ Globally, one‐third of adults may have reduced health literacy.^108^ Lower levels of health literacy in pregnancy are associated with unhealthy behaviors.^109^ Dietary advice in pregnancy should therefore be practical, implementable, and communicated clearly, using plain and simple language.^110^ The FIGO Nutrition Checklist is a tool that can be used to support this process. It is available online and free to download at: https://www.figo.org/news/figo‐nutrition‐checklist. A digital version of the FIGO Nutrition Checklist is also under development, which will allow for wider access of the resource through mobile or other electronic devices.^111^ Mobile health technologies provide easy access to information and tools, they are highly acceptable to women, and are especially useful for those with lower socioeconomic status, younger age, or raised body mass index.^112^ The FIGO Nutrition Checklist can also be used to assess response to a dietary intervention in pregnancy and appropriate core outcomes for nutritional studies are being developed.^113^ Dietary interventions also show promise in terms of cost‐effectiveness.^114^ For further insights on how the FIGO Nutrition Checklist can be used, see Figures 2, 3, 4. Future work will be to review this evidence and adapt the FIGO Nutrition Checklist as required.

## 13 CONCLUSION

Using the FIGO Nutrition Checklist supports identification of nutritional deficiencies in women and girls that can be addressed. The resource can be adapted and is suitable for use across a range of health settings, world regions, and throughout the life course.

## AUTHOR CONTRIBUTIONS

Sarah Louise Killeen and Niamh Donnellan wrote the manuscript with contributions from all other authors. All authors contributed to and reviewed the final manuscript.

## ACKNOWLEDGMENTS

Open access funding provided by IReL.

## CONFLICT OF INTEREST

Cynthia Maxwell reports grants from the Canadian Institutes for Health Research and the Crohns and Colitis Foundation of Canada. Harold David McIntyre reports honoraria for lectures from Phillips Health Care, Mead Johnson (China), and Diabetes Ireland. Sharleen O'Reilly reports research grants from the European Commission Horizon 2020, National Health and Medical Research Council of Australia, Health Research Board Ireland, Al Qasimi Foundation, and University of Sharjah. Lina Bergman reports research funds from Thermo Fischer, Roche, and Perkin Elmer and payment from Homburg and Partner. Ronald Ma reports research support from AstraZeneca, Bayer, Novo Nordisk, Pfizer, and Tricida Inc. Other authors have no conflicts of interests to declare.

## Supporting information


Figure S1
