# Supplementary figures and images for: Using FIGO Nutrition Checklist counselling in pregnancy: A review to support healthcare professionals
Source: Int J Gynaecol Obstet. 2023 Jan 12;160(Suppl 1):10–21. doi: 10.1002/ijgo.14539 (PMC10108324; doi:10.1002/ijgo.14539)

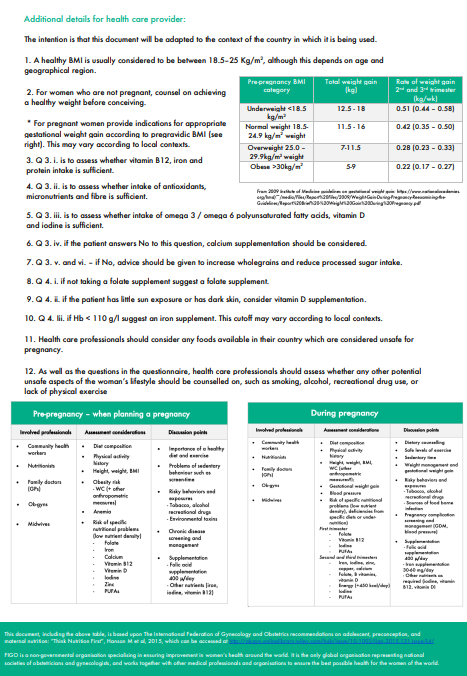

Supplement: Supplementary file 1 — Figure S1 [file IJGO-160-10-s001.png]
